# Supplementary material for: Effects of the Anti-Tumorigenic Agent AT101 on Human Glioblastoma Cells in the Microenvironmental Glioma Stem Cell Niche
Source: Int J Mol Sci. 2021 Mar 30;22(7):3606. doi: 10.3390/ijms22073606 (PMC8037174; doi:10.3390/ijms22073606)

Original images for Blots (supplementary to Figure 4)

Native cells stimulated with stem-like cell conditioned media (cm) in relation to the respective controls (m)

U251MG N=1

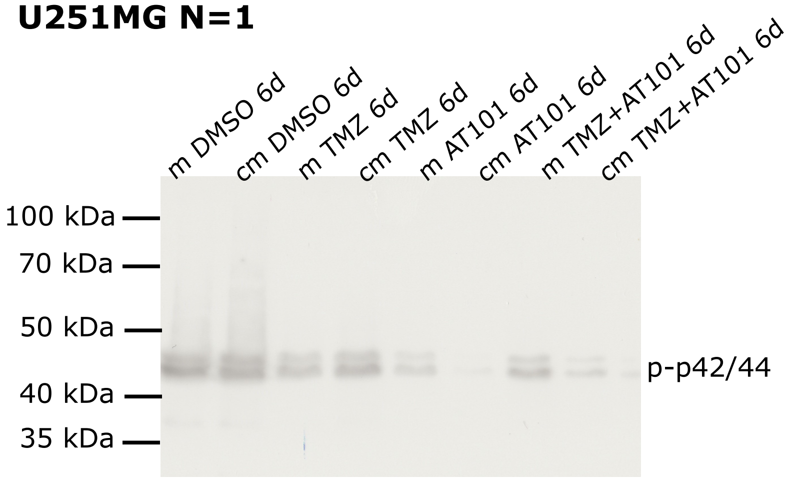

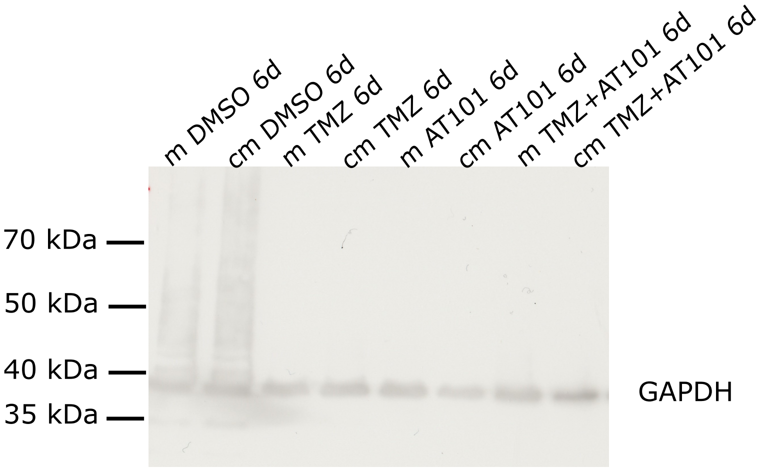

U251MG N=2

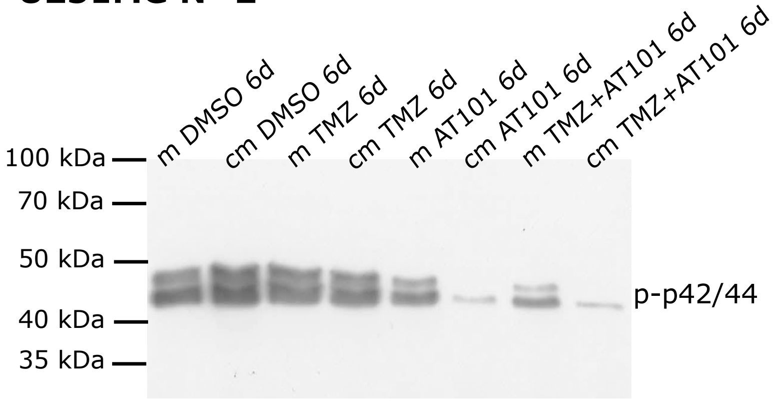

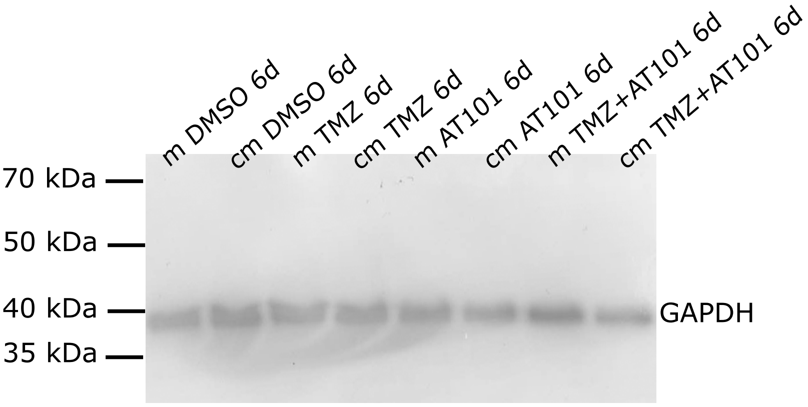

U87MG N=1

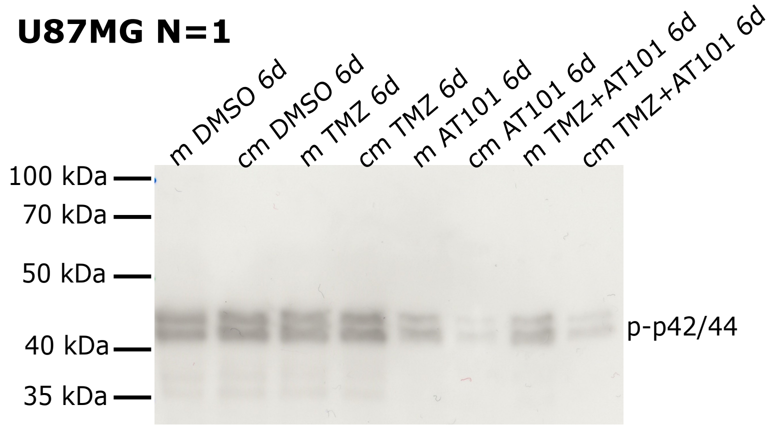

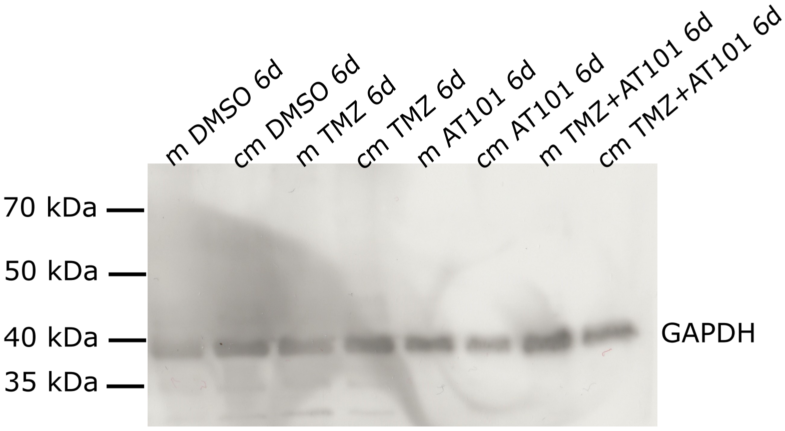

U87MG N=2

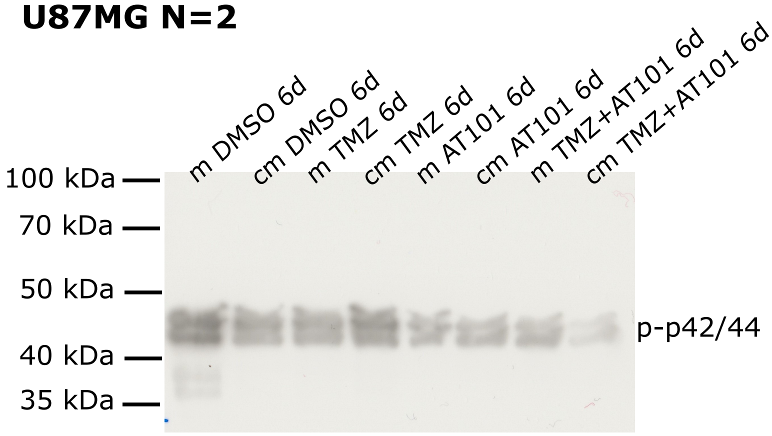

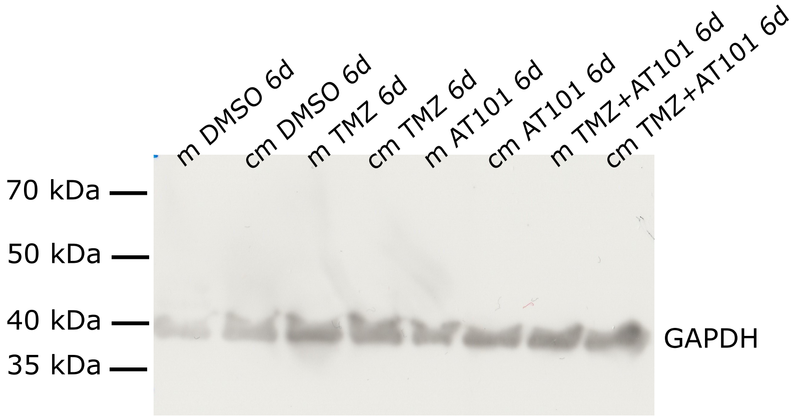

Supplement: Supplementary file 1 [file ijms-22-03606-s001.zip › Figure S1.pdf]
