# Supplementary material for: Effects of the Anti-Tumorigenic Agent AT101 on Human Glioblastoma Cells in the Microenvironmental Glioma Stem Cell Niche
Source: Int J Mol Sci. 2021 Mar 30;22(7):3606. doi: 10.3390/ijms22073606 (PMC8037174; doi:10.3390/ijms22073606)
Supplement: Supplementary file 1 [file ijms-22-03606-s001.zip › Figure S2.pdf]

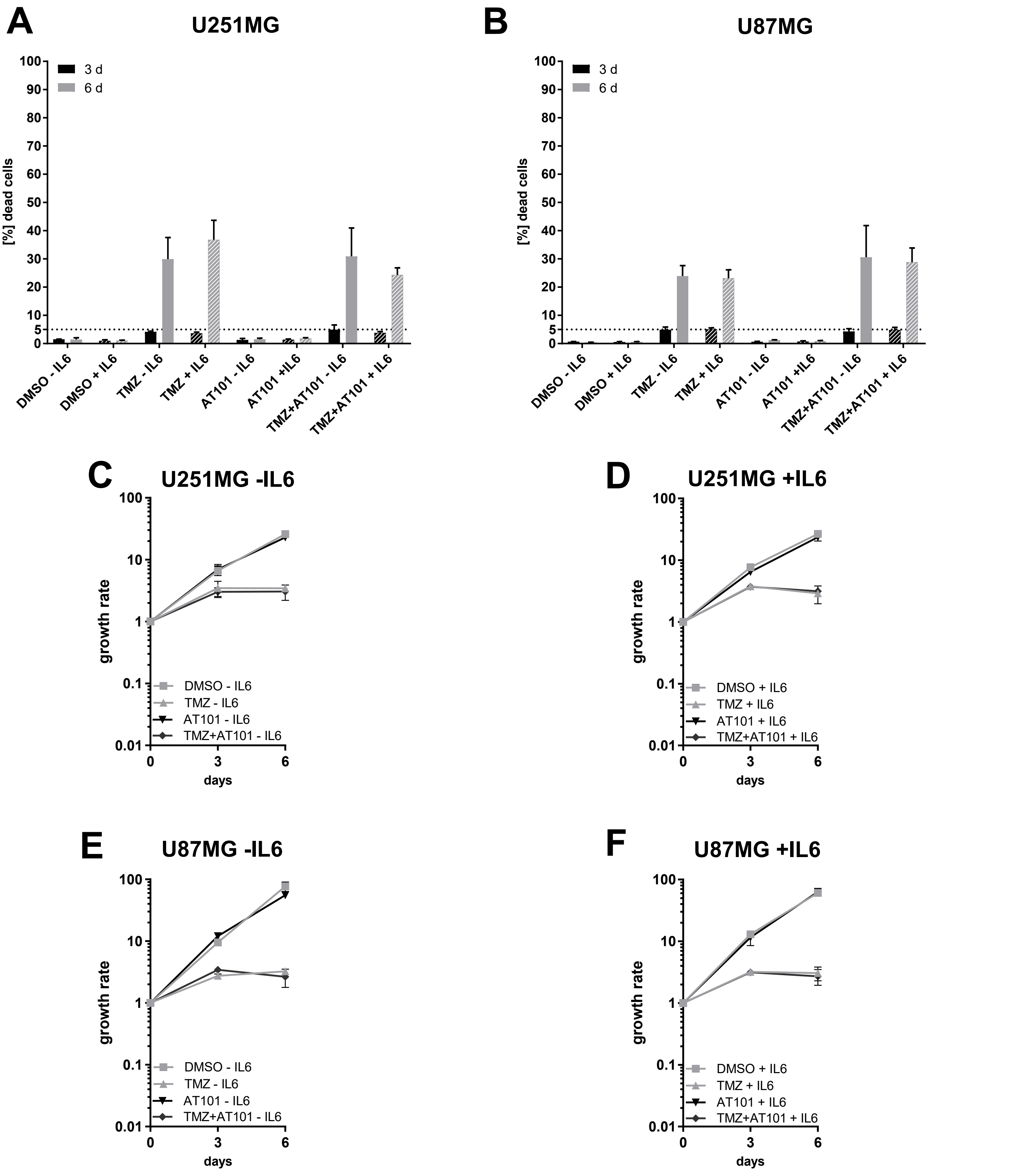

Figure S2: Stimulation with interleukin-6 yielded no effect on the survival and proliferation of native U251MG and U87MG cells. The different cell lines were stimulated with normal growth medium (DMEM plus 10 % fetal bovine serum) containing DMSO (dimethyl sulfoxide = control), temozolomide (TMZ) [50 µM], AT101 [5 µM], or a combination of TMZ [50 µM] and AT101 [5 µM], all with or without human recombinant interleukin-6 (IL6) [10 ng/ml]. A cytotoxicity assay was carried out after 3 and 6 days, respectively, revealing death rates of native U251MG (A) and U87MG (B) cells. Cell counts were determined on day 0, 3 and 6 of stimulation. Growth rates were calculated as n-fold amount of alive cells on day 3 and 6 compared to day 0 of stimulation for both U251MG and U87MG cells incubated with medium without IL6 (C and E) or medium with IL6 (D and F). Error bars correspond to the standard deviation, n = 2.
